# Oxidative Stress Compromises Lymphocyte Function in Neonatal Dairy Calves

**DOI:** 10.3390/antiox10020255

**Published:** 2021-02-07

**Authors:** Wilmer Cuervo, Lorraine M. Sordillo, Angel Abuelo

**Affiliations:** Department of Large Animal Clinical Sciences, College of Veterinary Medicine, Michigan State University, East Lansing, MI 48824, USA; cuervowi@msu.edu (W.C.); sordillo@msu.edu (L.M.S.)

**Keywords:** immune function, redox balance, oxidant status, lymphocyte, dairy cattle

## Abstract

Dairy calves are unable to mount an effective immune response during their first weeks of life, which contributes to increased disease susceptibility during this period. Oxidative stress (OS) diminishes the immune cell capabilities of humans and adult cows, and dairy calves also experience OS during their first month of life. However, the impact that OS may have on neonatal calf immunity remains unexplored. Thus, we aimed to evaluate the impact of OS on newborn calf lymphocyte functions. For this, we conducted two experiments. First, we assessed the association of OS status throughout the first month of age and the circulating concentrations of the cytokines interferon-gamma (IFN-γ) and interleukin (IL) 4, as well as the expression of cytokine-encoding genes *IFNG, IL2, IL4,* and *IL10* in peripheral mononuclear blood cells (PBMCs) of 12 calves. Subsequently, we isolated PBMCs from another 6 neonatal calves to investigate in vitro the effect of OS on immune responses in terms of activation of lymphocytes, cytokine expression, and antibody production following stimulation with phorbol 12-myristate 13-acetate or bovine herpesvirus-1. The results were compared statistically through mixed models. Calves exposed to high OS status in their first month of age showed higher concentrations of IL-4 and expression of *IL4* and *IL10* and lower concentrations of IFN-γ and expression of *IFNG* and *IL2* than calves exposed to lower OS. In vitro, OS reduced lymphocyte activation, production of antibodies, and protein and gene expression of key cytokines. Collectively, our results demonstrate that OS can compromise some immune responses of newborn calves. Hence, further studies are needed to explore the mechanisms of how OS affects the different lymphocyte subsets and the potential of ameliorating OS in newborn calves as a strategy to augment the functional capacity of calf immune cells, as well as enhance calves’ resistance to infections.

## 1. Introduction

Neonatal dairy calf morbidity and mortality risks associated with infectious diseases have been reported to be high in several countries [1,2,3]. A major factor contributing to disease susceptibility in the neonatal stage is calves’ inability to mount an effective immune response against pathogens [4]. This is, in part, attributed to the lower capacity of calf lymphocytes to respond to stimuli compared to lymphocytes from mature animals [4,5,6,7]. Critical functions of lymphocytes that are altered during the neonatal period in calves include, among others, activation capacity, cytokine production, and antibody production [8,9,10].

In humans, compelling evidence in the last three decades shows that changes in redox balance (RB) result in decreased lymphocyte responses to stimuli, particularly in T lymphocytes [8,9,10]. Redox balance reflects the equilibrium between the concentration of pro-oxidants and the availability of antioxidant defenses [11]. Excessive accumulation of pro-oxidants such as reactive oxygen and nitrogen species (RONS) can lead to disruption of cell membrane and damage to proteins, lipids, and DNA in a process known as oxidative stress (OS) [12,13,14]. There is also evidence in transition dairy cows indicating a negative effect of OS on immune responses [12,13,14]. Recent reports showed more dramatic RB changes during calves’ first month of life than in transition cows [15,16,17]. However, the impact that OS has on neonatal calf immunity remains unexplored. Thus, we hypothesized that OS conditions could result in compromised lymphocyte functions in neonatal calves. To test this hypothesis, we conducted two experiments in this study. The first experiment aimed at determining the association between RB and lymphocyte cytokine expression in dairy calves throughout the first month of life. The second experiment’s objective was to determine in vitro the impact of OS on selected calf lymphocyte functions relevant to the immune response to pathogens.

## 2. Materials and Methods

### 2.1. Experiment 1: Association of Redox Balance and Cytokine Expression in Neonatal Calves

#### 2.1.1. Animals and Blood Samples

For this experiment, blood samples collected for a previous study were utilized [18]. In short, 12 Holstein–Friesian calves (7 female, 5 male) were blood sampled weekly via jugular venipuncture for the first month of life (7 ± 0.8, 14 ± 1.0, 20 ± 0.8, and 29 ± 0.8 days of age). Blood was collected into plain vacuum tubes (BD Vacutainer; Becton, Dickinson and Company, Plymouth, UK) for serum collection. Blood was allowed to clot at room temperature, and sera were subsequently harvested after centrifugation at 2000× *g* for 20 min at 4 °C, aliquoted, and stored at −80 °C pending analysis within 2 months of collection. Blood from each calf was also collected in EDTA vacuumed tubes (Vacuette K3EDTA; Greiner Bio-One GmbH, Kremsmünster, Austria), immediately stored on crushed ice, and transported to the laboratory for isolation of peripheral blood mononuclear cells (PBMCs). The husbandry management of these calves is detailed in the study of the origin [18].

#### 2.1.2. Determination of Redox Balance

Systemic RB was assessed as previously described [19]. A commercially available fluorometric assay (OxiSelect In Vitro ROS/RNS Assay Kit; Cell Biolabs Inc., San Diego, CA, USA) was used to measure RONS in the serum as a marker of oxidant production. In brief, a specific probe was added to and reacted with free radicals within the sample, yielding a fluorescent product. The reported values represent relative fluorescent units normalized per microliter of sample.

The serum antioxidant potential (AOP) was measured using the Trolox equivalent antioxidant capacity, as previously described [20]. Briefly, the AOP of a sample was expressed as the equivalence of a known Trolox (synthetic vitamin E analog) standard (Sigma-Aldrich, St. Louis, MO, USA) concentration, resulting in a similar reduction of the generated radical 2,2′-azino-bis-3-ethylbenzothiazoline-6-sulfonic acid (Sigma, St. Louis, MO, USA) determined using the standard curve.

Redox balance was assessed as the ratio of pro-oxidant to total antioxidant defenses (RONS/AOP), namely the oxidant status index (OSi), as it accurately detects changes in RB during the transition period of dairy cattle [12]. An increase in the ratio suggests a higher risk for OS due to increased pro-oxidant production or defensive antioxidant depletion.

#### 2.1.3. Determination of Cytokine Expression

The expression of selected cytokines was quantified at both the protein and gene level. The concentrations of interferon-gamma (IFN-γ) and interleukin (IL) 4 in calf plasma were quantified using commercial ELISA assays (IFN-γ: IFN gamma Bovine Uncoated ELISA Kit, IL-4: Bovine IL-4 ELISA Kit; Invitrogen, Carlsbad, CA, USA). All samples were analyzed in duplicate. The intra- and inter-assay CVs were 5.6 and 7.8%, as well as 6.3 and 8.6% for the IFN-γ and IL-4 ELISA, respectively.

The mRNA expression of interferon-gamma (*IFNG*), *IL2*, *IL4*, and *IL10* genes in the calves’ PBMCs was also determined using real-time quantitative reverse–transcription PCR. For this, EDTA collected samples were centrifuged at 1300× *g* for 10 min at 4 °C, and the buffy coat containing the PBMCs was collected. A commercial isolation and purification kit, including on-column digestion of DNA, was used to isolate RNA from the PBMC samples (QIAamp RNA Blood Mini Kit and RNase-Free DNase Set; Qiagen, Hilgen, Germany). Isolated RNA samples were subject to an initial quality control measurement. Briefly, samples were quantitated using the Invitrogen Qubit and associated chemistry, which incorporates an RNA-specific fluorescent dye (Invitrogen, Carlsbad, CA, USA). The RNA’s integrity was measured using the Agilent Bioanalyzer 2100 microfluidics device (Agilent Technologies, Waldbronn, Germany). All samples had RNA integrity number values between 8 and 10, and the RNA concentration ranged from 34.5 to 389.1 ng/µL. RNA was stored at −80 °C pending analysis within 3 months.

For cDNA synthesis, up to 500 ng of RNA was reverse-transcribed (SuperScript III First-Strand Synthesis SuperMix, Life Technologies, Thermo Fisher Scientific, Waltham, MA, USA) and stored at −20 °C until analysis. Peptidylprolyl isomerase A (*PPIA*) was chosen as the endogenous control gene, as previously recommended [21]. Bovine-specific primer-probe sets with exon spanning probes were purchased for the control gene and all genes of interest (*IFNG*: Bt03212723, *IL2*: Bt03217368, *IL4*: Bt03211897, *IL10*: Bt03212727; TaqMan Gene Expression Assays, Applied Biosystems, Thermo Fisher Scientific, Waltham, MA, USA). Real-time quantitative reverse-transcription PCR was performed using a 2-fold dilution of cDNA at 10% of the final reaction volume. Each sample was analyzed in triplicate using a C1000 thermal cycler with real-time system CFX96’ (Bio-Rad, Hercules, CA, USA) and 2× master mix (TaqMan Gene Expression Master Mix, Applied Biosystems, Thermo Fisher Scientific, Waltham, MA, USA). The PCR protocol consisted of denaturation at 95 °C for 10 min, followed by 40 cycles of 95 °C for 15 s (denaturation) and 1 min at 60 °C (annealing and extension). For the calibration reference sample, cDNA obtained from PMBCs of a 148 days-in-milk dairy cow were used, as a low degree of OS is expected at this stage of lactation [22]. Results were analyzed using the comparative quantification algorithms-standard curve method (ΔΔCt method). Results are expressed as relative quantity (RQ = 2^−ΔΔCt^).

#### 2.1.4. Statistical Analyses

**Figure 1:**
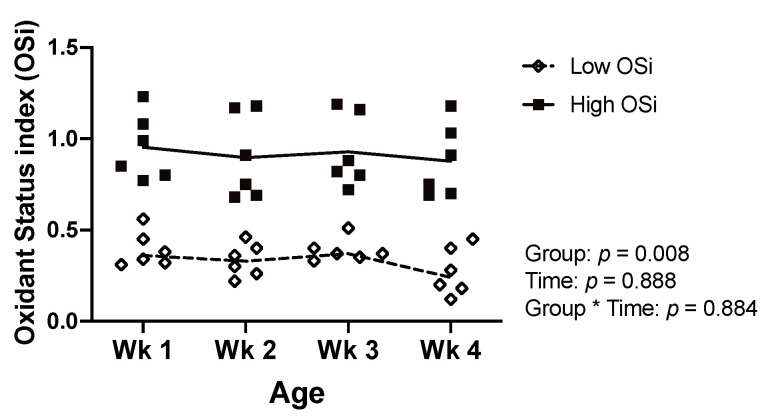
Values of the oxidant status index (OSi) of the 12 calves of Experiment 1 throughout the first 4 weeks (wk) of age according to the OSi group. Data are presented as individual values with lines connecting the means.

All statistical analyses were completed using JMP Pro 12 (SAS Institute Inc., Cary, NC). To create two groups of calves with contrasting degrees of RB, calves were classified ex-post based on their OSi values. For this, we calculated the average OSi for each calf based on the weekly determinations. Calves with a mean OSi below and above the median of the averages were placed in the low (*n* = 6) and high (*n* = 6) OSi groups, respectively. The resulting groups had a similar gender distribution (2 and 3 males in the low and high OSi group, respectively) and statistically significant OSi values as assessed via a mixed model with repeated measures (Figure 1).

Mixed models were built for the outcome variables serum concentration of IFN-γ and IL-4, as well as the RQ of *IFNG*, *IL2*, *IL4*, and *IL10*. Fixed effects included time (sampling week 1, 2, 3, or 4), calf RB group (high vs. low OSi), and the time × group interactions. Calf identification and sex were the random effects. For repeated measures, 5 covariance structures were tested (unstructured, autoregressive 1, variance components, compound symmetry, and Toeplitz), and the one resulting in the lowest Akaike information criterion was chosen. The degrees of freedom were approximated with the Kenward–Roger method. Model assumptions were assessed by evaluating homoscedasticity and normality of residuals. Tukey’s honest significance test was used for post hoc pairwise comparisons. Statistical significance was declared at *p* < 0.05.

### 2.2. Experiment 2: Impact of Oxidative Stress on Immune Responses of Neonatal Calves In Vitro

#### 2.2.1. Animals and Housing

In the second experiment, 6 Holstein female calves from a commercial dairy farm (Elsie MI, USA) were blood sampled at the age of 4 days. All calves were born from eutocic births with no or little assistance. Immediately after birth, calves received an oral vaccine against diarrhea pathogens (Calf-Guard, Zoetis Services, Parsippany, NJ, USA), an intranasal vaccine against respiratory pathogens (Bovilis Nasalgen 3, Merck Animal Health, Madison, NJ, USA), and 3 mL of a vitamin E and selenium complex subcutaneously (MU-SE, Merck Animal Health). After 30 min, calves received 4 L of warmed frozen colostrum with a Brix > 21% via orogastric tube and another 2 L of colostrum approximately 6 h later. Calves were housed in pairs in in-house stalls and fed 3 L of milk replacer (Cow’s Match, Land O’Lakes Inc., Arden Hills, MN, USA) 3 times/day with water available ad libitum.

#### 2.2.2. PBMC Isolation and Culture

A blood sample (50 mL) was collected from each animal via jugular venipuncture into tubes containing acid-citrate-dextrose (Becton, Dickinson and Company, Franklin Lakes, NJ, USA). PBMCs were isolated using a Ficoll-paque (Sigma, St. Louis, MO, USA) gradient, as previously described [23]. The PBMCs isolation procedure yielded viability greater than 93% as determined by trypan blue exclusion (Sigma, St. Louis, MO, USA) using an automated cell counter (Countess II FL, Life Technologies, Carlsbad, CA, USA). Following isolation, PBMCs were suspended on RPMI media with 10% fetal bovine serum and 2 mM L-glutamine (Gibco Laboratories, Gland Island, NY, USA), cultured in 6-well plates at a concentration of 2 × 10^6^ cells/mL, and assigned to one of 6 treatments:

1. AAPH: A treatment with 3 mM of the free radical generator 2,2′-Azobis(2-amidinopropane) dihydrochloride (AAPH), according to [24], to create OS conditions in vitro.
2. BHV-1: A viral challenge using bovine herpesvirus 1 (BHV-1 Colorado Strain, provided by the National Veterinary Services Laboratories, Animal and Plant Health Inspection Service, United States Department of Agriculture) at 8 × 10^4^ TCID_50_ per 1 × 10^6^ cells, according to [25].
3. PMA: A mitogenic stimulation with phorbol 12-myristate 13-acetate (PMA) and Ionomycin (Sigma, St Louis, MO) at 10 ng/mL and 1 µg/mL, respectively [26].
4. PMA + AAPH: The combination of PMA and AAPH.
5. BHV-1 + AAPH: The combination of BHV-1 and AAPH.
6. CON: PBMCs suspension on RPMI media as the negative control.

All samples were run in triplicate and incubated during 12 h at 37 °C and 5% CO_2._ The different concentrations of each treatment and incubation time were based on preliminary evaluations.

#### 2.2.3. Analytical Determinations

##### Viability

To confirm that lymphocyte functions were evaluated on viable cells, viability was measured using a commercial assay (Cell Viability Assay Cell-titter GLO; Promega, Madison, WI, USA). This assay captures the luminescent signal from the oxygenation of luciferin mediated by the amount of ATP present in the media, which is proportional to the number of metabolically active PBMCs. In short, following incubation, 100 μL of the 5 × 10^4^ PBMCs/mL suspension were extracted from the 6-well plate and plated in triplicate in a 96-well luminescence plate (Corning Costar, Tewksbury, MA, USA), 100 µL of the luciferin solution were added to each well and subsequently incubated for 30 min at room temperature. A positive control of *E. coli* lipopolysaccharide (2 µg/mL, LPS from E. coli 0111:B4, InvivoGen, San Diego, CA, USA) was included in this assay to ensure that the assay could detect decreases in viability. Results are expressed as relative luminescence, measured in a Gen5 plate reader (Synergy H1 Hybrid, Biotek, Winooski, VT, USA).

##### Oxidative Damage

The concentration of the isoprostane 8-iso prostaglandin F2α (8-iso-PGF2α) was determined as an indicator of lipid peroxidation in PBMCs because they are considered the gold standard indicator of OS in humans [27], and have also been previously used in cows to evaluate OS associated with coliform mastitis [28]. For this, the cell culture supernatant was harvested, acidified with an antioxidant-reducing agent composed of 50% methanol (Sigma), 25% ethanol (Sigma), and 25% of HPLC water (Sigma) with 0.9 mM butylated hydroxytoluene (Sigma), 0.54 mM EDTA (Sigma), 3.2 mM triphenylphosphine (Sigma), and 5.6 mM indomethacin (Sigma) to prevent further lipid peroxidation, flash-frozen in liquid nitrogen, and stored at −80 °C until analyses via liquid chromatography-tandem mass spectrometry, as previously described [29].

##### Lymphocyte Activation

The activation of lymphocytes in response to the treatments was measured by quantifying the expression of cluster of differentiation (CD) 69 via flow cytometry, as previously described in humans and cattle [30,31]. For this, PBMCs were recovered after incubation via centrifugation (450× *g*, 5 min) and washed three times with phosphate-buffered saline and 1% of bovine serum albumin solution, then 30 µg/mL of an anti-bovine CD69 monoclonal antibody (Cat No BOV2080/cell line KTSN7A, Monoclonal Antibody Center, Washington State University, Pullman, WA, USA) was added to every sample in a 96-well plate. After 15 min of incubation on ice, the plate was centrifugated at 670× *g* for 3 min and washed with horse serum-based buffer (Gibco Laboratories, Gland Island, NY, USA) 3 times. Subsequently, PBMCs were incubated with 15 µg/mL of a goat anti-mouse IgG1 secondary antibody labeled with Alexa Fluor 647 (Catalog # A-21240; Invitrogen, Carlsbad, CA, USA) in the dark on ice for 15 min. Then, samples were subjected to 2 cycles of centrifugation at 670× *g* for 3 min and washed with buffer without the horse serum. Non-specific binding of the secondary antibody was assessed by including the first wash buffer and second step reagent without cells. Samples were analyzed using a BD Accuri C6 flow Cytometer (BD Biosciences, San Jose, CA, USA). Lymphocytes were gated based on their forward and side scatter characteristics, registering 10.000 events within the selected gate. The activation index based on CD69 expression was estimated as described in Equation (1) [32]:

(1) $$CD69\;Expression\;Index=\frac{\left(\%\;stimulated\;CD69^{+}\;lymphocytes\right)\times \left(MFI\;of\;stimulated\;CD69^{+}\;lymphocytes\right)}{\left(\%\;nonstimulated\;CD69^{+}\;lymphocytes\right)\times \left(MFI\;of\;nonstimulated\;CD69^{+}\;lymphocytes\right)}\;\mathrm{MFI}\;=\;\mathrm{Mean}\;\mathrm{Fluorescent}\;\mathrm{Intensity}$$

##### Antigen-Specific Antibody Production

The production of immunoglobulins (Ig) against the in vitro viral challenge was measured via the quantification of anti-BHV-1 IgG in PBMC supernatant using a commercial ELISA assay (Bovichek BoHV-1 Antibody ELISA Test Kit; BioVet, Saint-Hyacinthe QC, Canada). The results are expressed as inhibition percentage according to the assay instructions (Equation (2)):

(2) $$\mathrm{Inhibition}\;\mathrm{percentage}\;\left(\%\right)\;=\;\left(1-\;\frac{\mathrm{OD}\;\mathrm{sample}}{\mathrm{mODneg}}\right)\times 100\;\mathrm{OD}=\;\mathrm{optical}\;\mathrm{density};\;\mathrm{mODneg}\;=\;\mathrm{mean}\;\mathrm{optical}\;\mathrm{density}\;\mathrm{value}\;\mathrm{of}\;\mathrm{the}\;\mathrm{negative}\;\mathrm{control}\;\mathrm{of}\;\mathrm{the}\;\mathrm{assay}.$$

##### Cytokine Production

Quantification of IFN-γ and IL-4 production was determined in the cell culture supernatant using commercial ELISA assays (IFN-γ: IFN gamma Bovine Uncoated ELISA Kit, IL-4: Bovine IL-4 ELISA Kit; Invitrogen, Carlsbad, CA, USA). All samples were analyzed in duplicate following the manufacturer’s instructions. The intra- and inter-assay CVs were 7.0 and 3.1, and 9.5 and 3.5% for the IFN-γ and IL-4 ELISAs, respectively.

##### Cytokine mRNA Expression

Messenger RNA was extracted from cultured lymphocytes (1.2 × 10^6^ cells/mL) using the RNeasy Plus Mini Kit (Qiagen, Hilgen, Germany ). The obtained mRNA concentration was quantified using a NanoDrop 1000 (Thermo Scientific, Wilmington, DE, USA). All samples had a 260/280 ratio between 1.9 and 2.1 with a range of RNA concentration between 0.52 and 1.15 μg/μL and were adjusted to a final concentration of 0.5 μg/μL. Subsequently, RNA was subjected to reverse transcription using oligo-dT primers (High-Capacity cDNA Reverse Transcription Kit with RNAse inhibitor—Applied Biosystems, Thermo Fisher Scientific, Waltham, MA, USA), and reverse transcriptase (MultiScribe™) according to the manufacturer’s recommendations. Aliquots of cDNA (1 μL/well) from each sample were included in triplicate with 9 μL/well of real-time PCR master mix (TaqMan™ Fast Advanced Master Mix, Applied Biosystems, Thermo Fisher Scientific, Waltham, MA, USA). mRNA from untreated lymphocytes obtained of a mature non-lactating dairy cow was used as the reference sample. Glyceraldehyde-3-phosphate dehydrogenase (*GAPDH*) was included as the endogenous control, as described by [33]. Bovine-specific primer probe sets with exon spanning probes were purchased for the control gene and all genes of interest (*IFNG*: Bt03212723, *IL2*: Bt03217368, *IL4*: Bt03211897, *IL10*: Bt03212724; *GADPH*: Bt03210913; TaqMan Gene Expression Assays, Applied Biosystems, Thermo Fisher Scientific, Waltham, MA, USA). Real-time PCR was performed in a 384 well-plate using a QuantStudio7 thermocycler (Applied Biosystems, Thermo Fisher Scientific, Waltham, MA, USA) using the TaqMan Gene Expression Assay (Thermo Fisher Scientific, Waltham, MA, USA). An initial cycle of incubation (50 °C for 2 min) was followed by 40 cycles of polymerase activation at 95 °C for 20 s, followed by a dual step with 1 sec of denaturation at 95 °C and 20 s annealing at 60 °C. RNase-DNase-free water was included as a negative control for each gene. Results were analyzed using the ΔΔCt method, expressed as RQ normalized to negative control (CON).

#### 2.2.4. Statistical Analyses

All statistical analyses were conducted using SAS, version 9.4 for Windows (SAS Institute Inc., Cary, NC). Mixed models were used to investigate the effect of treatment (AAPH, PMA, BHV-1, PMA + AAPH, BHV-1 + AAPH, and CON) on the evaluated immune functions (activation, cytokine production, expression, and antigen-specific antibody production). Each calf was included as a random effect to account for individual variability. The degrees of freedom were approximated with the Kenward–Roger method. Model assumptions were assessed by evaluating homoscedasticity and normality of residuals. To meet these assumptions, the data of 8-iso-PGF2α were log-transformed and presented back-transformed as geometric means. For the viability assay, differences between the control and experimental treatments were determined using Dunnett’s pairwise post hoc test. For the other measurements, differences among all treatments were detected using Tukey’s test. Data are reported as estimated means ± SEM, and statistical significance was declared at *p* < 0.05.

## 3. Results and Discussion

### 3.1. Association of Redox Balance and Cytokine Expression in Neonatal Calves

**Figure 2:**
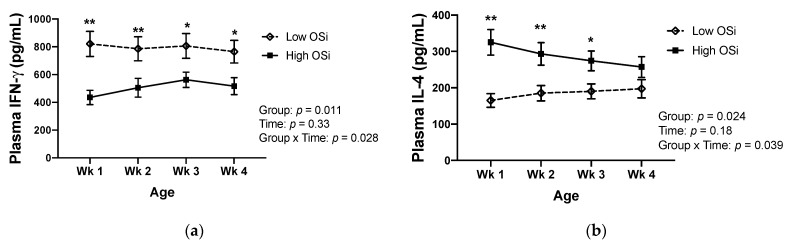
Changes in plasma concentration of (**a**) interferon-gamma (IFN-γ) and (**b**) interleukin (IL) 4 in calves throughout their first 4 weeks of life, according to their classification in the oxidant status index (OSi) groups (*n* = 6/group). The “low OSi” group includes the 6 calves with the lowest average OSi values based on weekly measurements. Results are expressed as estimated means ± SE. * *p* < 0.05, ** *p* < 0.01.

Redox balance throughout the first month of life was associated with differences in the circulating concentrations of IFN-γ and IL-4 in contrasting patterns (Figure 2). The plasma concentration of IFN-γ was significantly lower in calves exhibiting higher OSi throughout their first month of life. In contrast, significantly higher IL-4 plasma concentrations were detected in the higher OSi group in the first three weeks of life. At 4 weeks of age, the circulating concentration of IL-4 was still numerically greater in the high OSi group than the low OSi one, although the difference did not reach statistical significance (*p* = 0.072).

**Figure 3:**
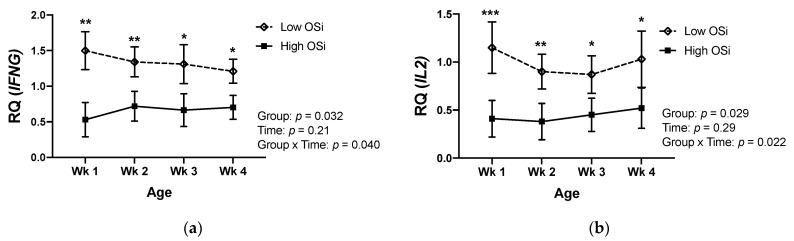

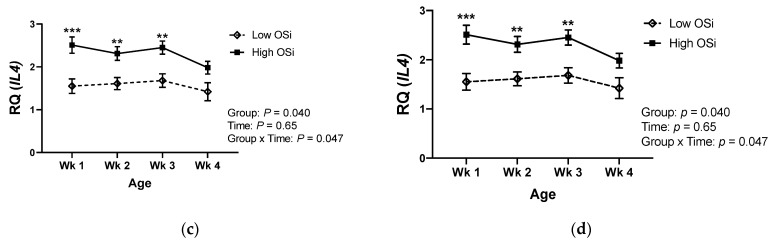
Relative mRNA expression of the genes (**a**) Interferon Gamma (*IFNG*), (**b**) Interleukin 2 (*IL2*), (**c**) Interleukin 4 (*IL4*), and (**d**) Interleukin 10 (*IL10*) in peripheral mononuclear blood cells of newborn calves with contrasting levels of redox balance, as assessed by the oxidant status index (OSi). The “low Osi” group includes the 6 calves with the lowest average OSi values based on weekly measurements. Data were analyzed using mixed models with repeated measures and Tukey’s honestly significant difference test. Results are expressed as estimated means ± SEM of relative quantity (RQ). * *p* < 0.05, ** *p* < 0.01, *** *p* < 0.001.

Similar to the differences observed in the circulating cytokine concentrations, gene expression of *IFNG* in PBMCs was lower in high OSi calves during their first month of life (Figure 3a). The same pattern was observed for the gene expression of *IL2* in those calves (Figure 3b). Conversely, mRNA expression of *IL4* and *IL10* was higher in calves with a higher OSi during the first 4 weeks of life.

We evaluated the association between calves’ RB and a selected number of cytokines due to their fundamental role in driving immune responses [34]. Our results showed for the first time that the RB of the calves was associated with differences in their profile of cytokines at both protein and gene expression levels. Interestingly, calves exposed to a more pro-oxidant RB (high OSi group) showed greater circulating concentrations of the Th2 cytokine IL-4, whereas the concentration of the Th1 cytokine IFN-γ was lower in this group. The mRNA expression of genes encoding cytokines showed a similar pattern, with greater Th2 (*IL4* and *IL10*) and lower Th1 (*IFNG* and *IL2)* expression in calves of the high OSi group. Thus, suggesting that calves exposed to a more pro-oxidant RB an increased Th2 and reduced Th1 response in comparison to the calves exposed to a lower pro-oxidant challenge. Previous studies have demonstrated that OS promotes a polarization of human T cell differentiation toward the Th2 phenotype [35,36], and our results suggest that this might also be the case in calves. However, other immune cell types are capable of synthetizing these cytokines and, therefore, the extent to which a higher pro-oxidant status results in increased Th2 cells needs to be further evaluated characterizing the different Th subsets and correlating the results with those of circulating cytokines. Nevertheless, a biased Th2 response characterizes the immune responses of neonatal calves during the first weeks of age, negatively impacting the immune memory capacity of these animals [4]. Thus, if RB also affects T cell differentiation in neonatal calves, strategies that manipulate RB during the first weeks of life could potentially be used as an avenue to change the immune responses of neonatal calves. 

### 3.2. Impact of Oxidative Stress on Immune Responses of Neonatal Calves In Vitro

#### 3.2.1. Viability and Isoprostane Concentration

**Figure 4:**
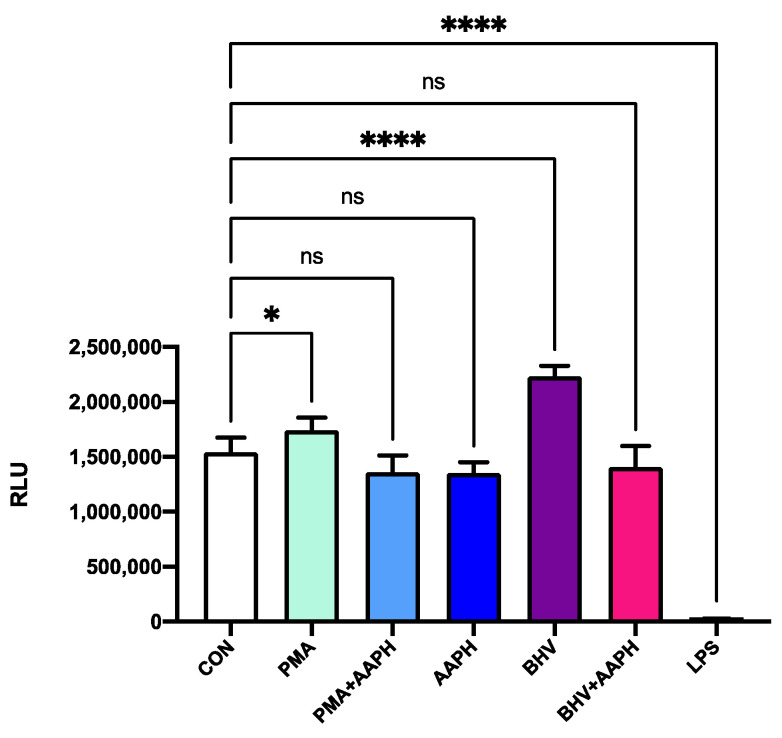
Viability of PBMCs after 12 h of incubation with the different treatments. Results are expressed as estimated means ± SEM of relative luminesce units (RLU). CON = Untreated peripheral mononuclear blood cells (PBMCs); PMA = PBMCs treated with 10 ng/mL phorbol 12-myristate 13-acetate (PMA) and 1 µg/mL ionomycin; PMA + AAPH = PBMCs treated with 10 ng/mL PMA, 1 µg/mL ionomycin, and 3 mM of 2,2′-Azobis(2-amidinopropane) dihydrochloride (AAPH); AAPH = PBMCs treated with 3 mM AAPH; BHV = PBMCs treated 8 × 10^4^ TCID_50_ Bovine Herpesvirus 1 per 1 × 10^6^ PBMCs; BHV + AAPH = PBMCs treated 8 × 10^4^ TCID_50_ Bovine Herpesvirus 1 per 1 × 10^6^ PBMCs and 3 mM AAPH; LPS = PBMCs treated with 2 µg/mL *E. coli* lipopolysaccharide (positive control). Differences between treatments and the control were assessed with Dunnett’s pairwise post hoc test. * *p* < 0.05, **** *p* < 0.0001, ns: *p* > 0.05.

**Figure 5:**
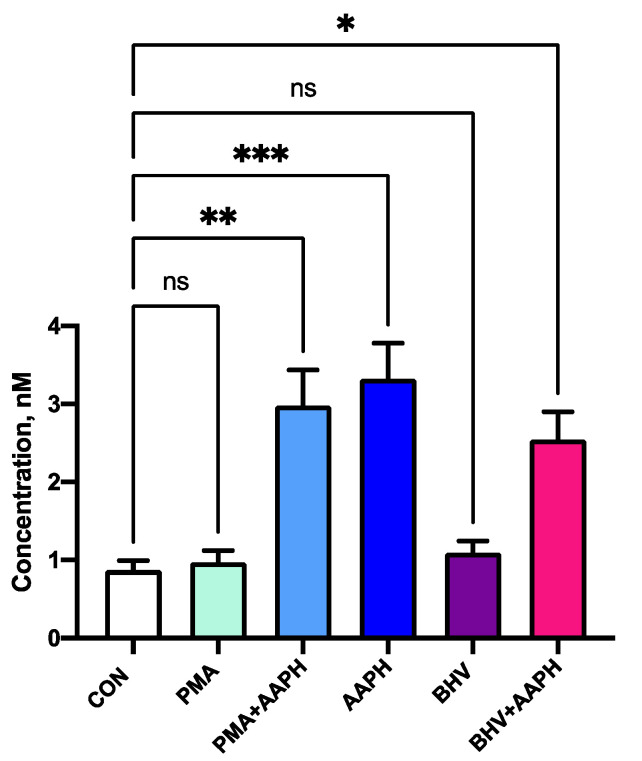
Concentration of 8-iso-Prostaglandin F2α in peripheral mononuclear blood cells (PBMCs) culture supernatant. Results are expressed as geometric estimated means ± SEM. CON = Untreated PBMCs; PMA = PBMCs treated with 10 ng/mL phorbol 12-myristate 13-acetate (PMA) and 1 µg/mL ionomycin; PMA + AAPH = PBMCs treated with 10 ng/mL PMA, 1 µg/mL ionomycin, and 3 mM of 2,2′-Azobis(2-amidinopropane) dihydrochloride (AAPH); AAPH = PBMCs treated with 3 mM AAPH; BHV = PBMCs treated 8 × 10^4^ TCID_50_ Bovine Herpesvirus 1 per 1 × 10^6^ PBMCs; BHV + AAPH = PBMCs treated 8 × 10^4^ TCID_50_ Bovine Herpesvirus 1 per 1 × 10^6^ PBMCs and 3 mM AAPH. Differences between treatments and the control were assessed with Dunnett’s pairwise post hoc test * *p* < 0.05, ** *p* < 0.01, *** *p* < 0.001.

None of the in vitro treatments used in this study resulted in a significant decrease in viability after 12 h of incubation (Figure 4). However, the use of AAPH at 3 mM for 12 h resulted in greater 8-iso-PGF2α concentrations in the cell culture media (Figure 5), indicating an increase in the oxidative damage to cell membrane phospholipids. In the viability results, there was a slight increase in luminescence detected in the stimulus treatments (PMA and BHV), but this could be attributed to the increase in ATP that characterizes the first stages of immune cell activation [37].

Thus, the in vitro conditions used in this experiment were capable of creating OS conditions that increased lipid peroxidation while maintaining the viability of cultured PBMCs. Accordingly, differences observed in the cells subjected to incubation with AAPH are not due to a decrease in the number of viable cells but likely represent changes in cell function associated with OS conditions.

#### 3.2.2. Activation of Lymphocytes

**Figure 6:**
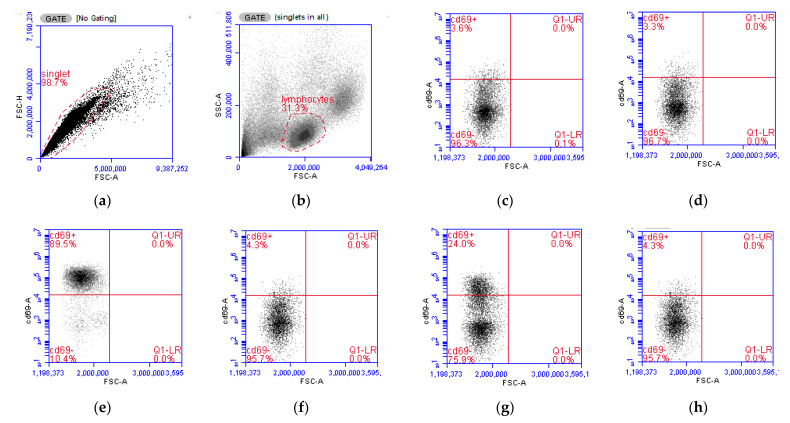

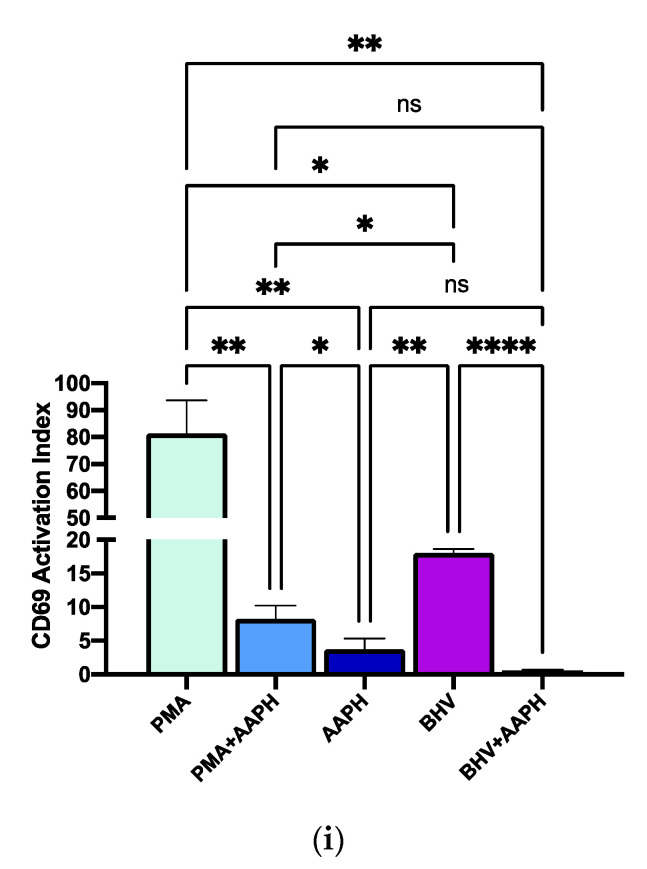
Flow cytometry analysis of lymphocyte populations from isolated peripheral mononuclear blood cells (PBMCs). (**a**). Gating strategy for singlets. (**b**) Gating strategy for lymphocytes based on forward scatter (FSC-A) and side scatter (SSC-A) characteristics. (**c**) CD69+ expression in untreated PBMCs, on the X axis FSC-A indicating cell size and on the Y axis the evaluated CD69 dye. (**d**) CD69 expression under 2,2′-Azobis(2-amidinopropane) dihydrochloride (AAPH) treatment. (**e**) CD69 expression under 10 ng/mL phorbol 12-myristate 13-acetate (PMA) treatment. (**f**) CD69 expression under PMA + AAPH. (**g**) CD69 expression under Bovine Herpesvirus 1 (BHV-1) stimulation. (**h**) CD69 expression under BHV-1 + AAPH. (**i**) Lymphocytes’ activation Index according to the CD69 expression index (Equation (2)). PMA = PBMCs treated with 10 ng/mL PMA and 1 µg/mL ionomycin; PMA + AAPH = PBMCs treated with 10 ng/mL PMA, 1 µg/mL ionomycin, and 3 mM AAPH; AAPH = PBMCs treated with 3 mM AAPH; BHV = PBMCs treated 8 × 10^4^ TCID_50_ BHV-1 per 1 × 10^6^ PBMCs; BHV-1 + AAPH = PBMCs treated 8 × 10^4^ TCID_50_ BHV-1 per 1 × 10^6^ PBMCs and 3 mM AAPH. Results are expressed as estimated means ± SEM and were compared with Tukey’s test. * *p* < 0.05, ** *p* < 0.01, **** *p* < 0.0001, ns: *p* > 0.05.

In this experiment, two immune cell activators with different mode of actions were used to study the impact of OS conditions on in vitro immune responses. PMA activates protein kinase C directly in the cytoplasm, being, therefore, independent of surface receptor stimulation [38]. Conversely, the activation mediated by BHV-1 relies on the presentation of the virus by antigen-presenting cells to the surface receptors of T cells [39]. In our study, the stimulation of PBMCs with either PMA or BHV-1 resulted in a significant increase in lymphocyte activation (Figure 6). However, when PBMCs were stimulated under OS conditions, the CD69 activation index of lymphocytes was significantly reduced (*p* < 0.001) in both studied stimuli.

The expression of the membrane protein CD69 was used as a marker of activation of lymphocytes as it is considered one of the earliest lymphocytes’ activation markers to be detected, with gene expression upregulation and membrane expression being detected as early as 2 and 7 h post-stimulation [30,40].

The stimuli included in this study followed different routes, thus, contrary to the APC-mediated BHV-1 stimulation, PMA treatment bypassed the canonical activation signaling pathway through the receptor and directly flooded the cell with calcium, promoting a rapid and robust activation of MAP kinase pathways [39,41]. CD69 expression in T lymphocytes is induced primarily via RAS-dependent Protein kinase C activation [42,43]. RAS is a redox-sensitive protein [44], and there is evidence that OS changes amino acid residues in RAS that result in altered CD69 expression [45]. Similarly, protein kinase C is also sensitive to RB changes and OS conditions that can produce structural modifications interfering with the activity of this kinase [46]. Moreover, the observed reduced lymphocyte activation could also be attributed to structural changes in the cell membrane due to oxidative damage. For example, in vitro exposure to pro-oxidants resulted in altered T cell receptor structure, stability, and aggregation and binding capacity [47,48]. Similarly, OS caused a reduction of the membrane expression of immune receptors in cultured human macrophages, resulting in decreased activation and phagocytic capacity of macrophages [49]. Ultimately, the observed decreased expression of CD69 in calf PBMCs stimulated under OS conditions in vitro suggest that OS can limit the efficiency of neonatal calves’ immune cells to initiate an immune response. 

#### 3.2.3. Antigen-Specific Antibody Production

**Figure 7:**
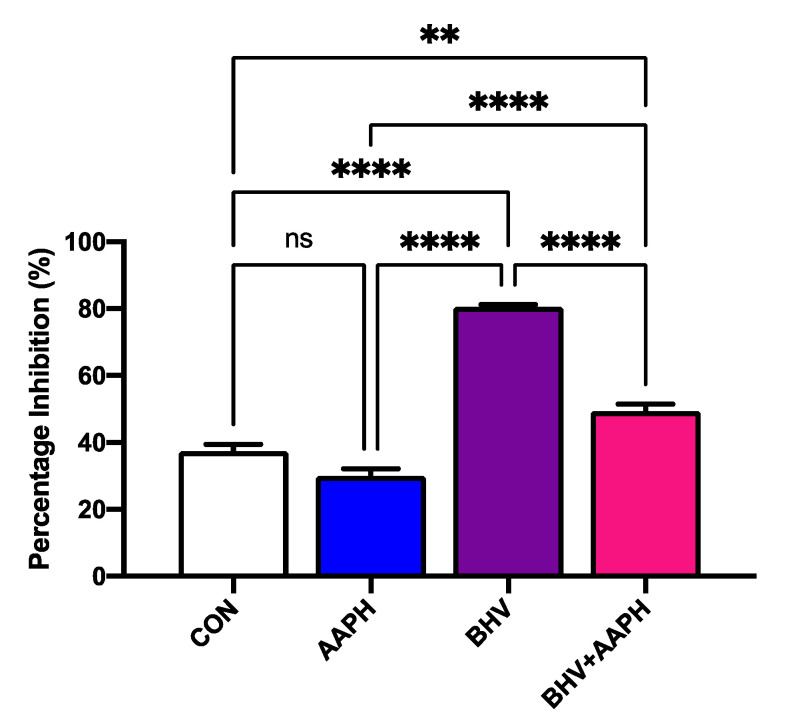
Antigen-specific antibody production across in vitro treatments expressed as estimated means ± SEM of inhibition percentage. CON = Untreated peripheral mononuclear blood cells (PBMCs); AAPH = PBMCs treated with 3 mM of 2,2′-Azobis(2-amidinopropane) dihydrochloride (AAPH); BHV = PBMCs treated 8 × 10^4^ TCID_50_ Bovine Herpesvirus 1 per 1 × 10^6^ PBMCs; BHV-1 + AAPH = PBMCs treated 8 × 10^4^ TCID_50_ Bovine Herpesvirus 1 per 1 × 10^6^ PBMCs and 3 mM AAPH. An increase in % inhibition means an increase in the amount of anti-BHV-1 IgG. Results were compared with Tukey’s test. ** *p* < 0.01, **** *p* < 0.0001, ns: *p* > 0.05.

The in vitro treatment with BHV-1 for 12 h resulted in a significant increase in antigen-specific antibody production when compared to untreated PBMCs (*p* < 0.0001; Figure 7). However, a significant reduction in the concentration of antibodies was detected when PBMCs were stimulated with the virus and AAPH (*p* < 0.0001). These results suggest that the lymphocytes’ capacity to synthesize antibodies in response to a pathogen, assessed here through immunoglobulin production against BHV-1, is negatively impacted when neonatal calves are under OS.

The production of antibodies against an antigen is the hallmark of the humoral immune response, requires the coordination of antigen presenting cells, T helper cells, and B cells, and plays an essential role in fighting infections [50]. However, the immune response of young calves is characterized by low concentrations of immunoglobulins [5]. Our results demonstrate that OS conditions reduce the ability of neonatal calf immune cells to synthesize immunoglobulins in response to a viral challenge. Thus, it is possible that the high levels of OS documented in calves during the first weeks of age [51,52,53] contribute to the low amount of immunoglobulins produced by calves. It is unclear, however, how OS negatively influences immunoglobulin production in these animals. On the one hand, OS could reduce the peptide presentation capacity of antigen-presenting cells, as documented in mice [54]. On the other hand, OS could directly influence the synthesis of immunoglobulins. In human B cells, OS induces endoplasmic reticulum stress, resulting in reduced immunoglobulin synthesis, folding, and secretion [55,56].

Similar to our results, the addition of a pro-oxidant to PBMCs from 13-month-old calves vaccinated against BHV-1 resulted in reduced cell antioxidant capacity and lower concentration of neutralizing antibodies against BHV-1 [57]. These results from animals with a mature immune system are comparable with our findings from calves with a naïve immune system. Thus, suggesting that OS can impair immunoglobulin production regardless of the level of maturity of the immune system of calves. Consequently, OS might be an underlying factor in calf disease susceptibility.

#### 3.2.4. Cytokine Protein and mRNA Expression

**Figure 8:**
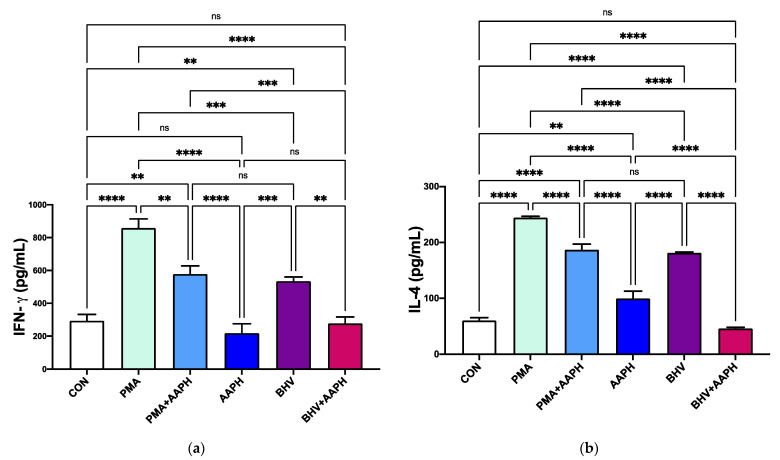
In vitro cytokine production of (**a**) interferon gamma (IFN-γ) and (**b**) interleukin 4 (IL-4). Results are expressed as estimated means ± SEM. CON = Untreated peripheral mononuclear blood cells (PBMCs); PMA = PBMCs treated with 10 ng/mL phorbol 12-myristate 13-acetate (PMA) and 1 µg/mL ionomycin; PMA + AAPH = PBMCs treated with 10 ng/mL PMA, 1 µg/mL ionomycin, and 3 mM of 2,2′-Azobis(2-amidinopropane) dihydrochloride (AAPH); AAPH = PBMCs treated with 3 mM AAPH; BHV = PBMCs treated 8 × 10^4^ TCID_50_ Bovine Herpesvirus 1 per 1 × 10^6^ PBMCs; BHV + AAPH = PBMCs treated 8 × 10^4^ TCID_50_ Bovine Herpesvirus 1 per 1 × 10^6^ PBMCs and 3 mM AAPH. Results were compared with Tukey’s test. ** *p* < 0.01, *** *p* < 0.001, **** *p* < 0.0001, ns: *p* > 0.05.

Both stimuli (PMA and BHV-1) increased in vitro the production of the two cytokines measured via ELISA (Figure 8), indicating an activation of the immune cells. Regardless of the stimuli, activation of PBMCs under OS conditions resulted in lower concentrations of cytokines being produced (*p* < 0.001).

**Figure 9:**
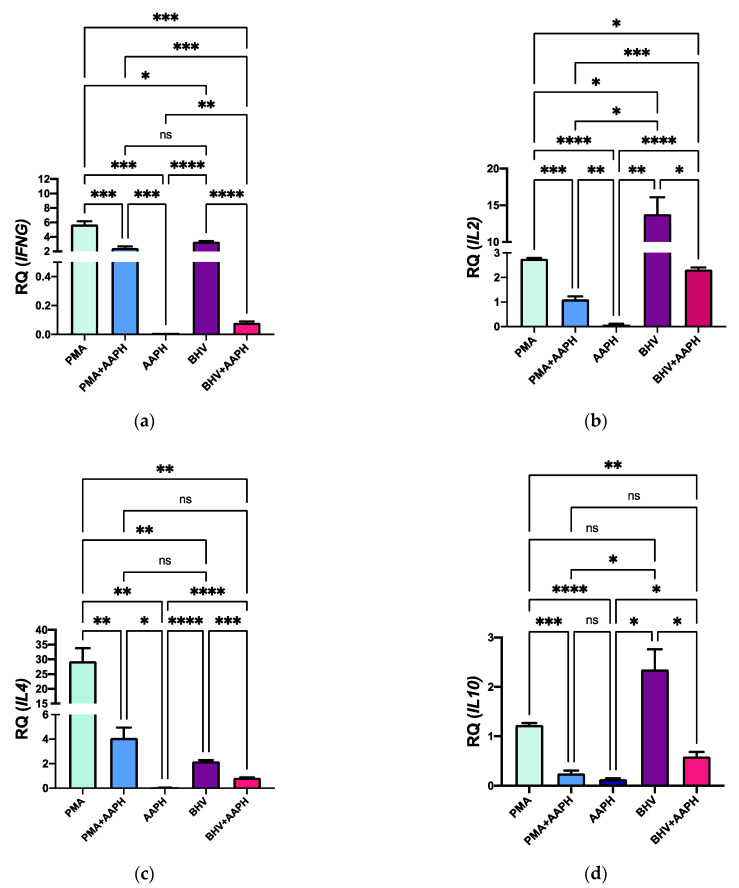
Relative expression of the genes (**a**) Interferon Gamma (*IFNG*), (**b**) Interleukin 2 (*IL2*), (**c**) Interleukin 4 (*IL4*), and (**d**) Interleukin 10 (*IL10*) in peripheral mononuclear blood cells (PBMCs) of newborn calves. Results are normalized to the gene expression of the negative control (CON). PMA = PBMCs treated with 10 ng/mL phorbol 12-myristate 13-acetate (PMA) and 1 µg/mL ionomycin; PMA + AAPH = PBMCs treated with 10 ng/mL PMA, 1 µg/mL ionomycin, and 3 mM of 2,2′-Azobis(2-amidinopropane) dihydrochloride (AAPH); AAPH = PBMCs treated with 3 mM AAPH; BHV = PBMCs treated 8 × 10^4^ TCID_50_ Bovine Herpesvirus 1 per 1 × 10^6^ PBMCs; BHV + AAPH = PBMCs treated 8 × 10^4^ TCID_50_ Bovine Herpesvirus 1 per 1 × 10^6^ PBMCs and 3 mM AAPH. Results are expressed as estimated means ± SEM of relative quantity (RQ). * *p* < 0.05, ** *p* < 0.01, *** *p* < 0.001, **** *p* < 0.0001, ns: *p* > 0.05.

In agreement with the results observed in cytokines’ expression at the protein level, OS conditions substantially downregulated expression of all the cytokines included in the study (Figure 9). Compared to CON, PMA-stimulated PBMCs exhibit an upregulated mRNA expression of *IFNG*, *IL2*, *IL4*, and *IL10* in 6, 27, 2.5, and 1-fold, respectively. However, stimulation of PBMCs with PMA under OS conditions reduced the mRNA expression of all the evaluated cytokine-encoding genes. A similar pattern was observed for BHV-1 stimulation, where the mRNA expression of all studied genes was reduced (*p* < 0.05) in PBMCs stimulated under OS conditions compared to those just challenged with the virus.

Taken together, our results demonstrate a decreased expression at both the protein and gene level of cytokines relevant for initiating an immune response in calf PBMCs. In our study, we are unable to attribute the changes in cytokine expression to specific populations of immune cells, as the experiments were conducted in PBMCs. However, previous studies in other species already showed alterations of cytokine production in lymphocytes under OS. In mice and human lymphocytes, for example, a simultaneous mitogen stimulation and oxidative challenge resulted on a lower production of IFN-γ, IL-2, and IL-4 [58,59,60]. These changes in cytokine production might be due to the effect of OS on cellular signaling. In human lymphocytes, high RONS concentrations affected the transcriptional regulator STAT3 [61], which is known to modulate the transduction of cytokines [62]. Moreover, the reduction in IL-2 gene and protein expression in response to an oxidant challenge in the absence of decreased PBMCs’ viability or changes in the activity of antioxidant enzymes supports that these changes are not due to cellular damage [47,63]. Furthermore, OS conditions influence a variety of redox-regulated signal transduction pathways modulating cytokines production in human and adult cattle [64,65]. Thus, it is possible that OS alter cytokine production at both transcriptional and post-transcription levels. This decrease in cytokine expression under OS conditions is in line with the other findings of this study and could be one of the factors that contribute to the decreased CD69 expression and antibody production observed in AAPH-treated PBMCs.

Interestingly, in Experiment 1, we observed contrasting expression of Th1 (IFN-γ and IL-2) and Th2 (IL-4 and IL-10) cytokines in association with changes in systemic RB, whereas in vitro OS reduced the expression of all cytokines in Experiment 2. These differences could be attributed to the fact that only differences in RB were assessed in Experiment 1, while in Experiment 2 we created oxidative damage in vitro, as evidenced by the higher rates of lipid peroxidation that resulted in increased 8-iso-PGF2α. Thus, it is possible that shifts in RB are able to modulate the immune response of calves, as shown in humans [35,36]. However, when the imbalance between pro-oxidant production and availability of antioxidants results in significant oxidative damage, the capability of the animals to mount an immune response is negatively affected. This highlights the need for accurately differentiating shifts in RB from OS. Changes in RB can be related to physiological processes [66], but is usually the oxidative damage that characterizes OS what leads to cellular dysfunction [67]. In dairy cattle, however, we currently lack the ability to accurately identify animals experiencing OS, as critical cut-off points for OS biomarkers are yet to be established [68,69]. Thus, limiting our ability to further understand the implications of redox biology in calves’ immune responses in vivo. Nevertheless, the results from this study clearly indicate an association between calves’ immunity and RB and OS. The results from this study are important because they provide the scientific underpinning that OS adversely impacts the functional capacity of calf lymphocytes. The immune functions assessed in the in vitro study are critical for fighting infections or vaccine responsiveness. Thus, OS might be a contributing factor to the increased disease morbidity observed in neonatal calves. Future studies can now explore the mechanisms of how OS compromises lymphocyte function in newborn calves and the extent to which supplementing calves with antioxidants might improve the compromised immune responses of neonatal dairy calves.

## 4. Conclusions

Collectively, our results demonstrate, for the first time, that OS can compromise some functions of lymphocytes obtained from newborn calves. Further studies are now needed to characterize the mechanisms of how OS impacts lymphocyte function in newborn calves, including evaluating its effects in the different lymphocyte subsets. Ultimately, the potential of ameliorating OS in newborn calves as a strategy to augment the functional capacity of calf immune cells and consequently enhance calves’ resistance to infections warrants further research.

## Data Availability

All data generated or analyzed during this study are included in this published article.
